# Parameter estimation of Muskingum model using grey wolf optimizer algorithm

**DOI:** 10.1016/j.mex.2021.101589

**Published:** 2021-11-23

**Authors:** Reyhaneh Akbari, Masoud-Reza Hessami-Kermani

**Affiliations:** Department of Civil Engineering, Shahid Bahonar University of Kerman, Kerman, Iran

**Keywords:** Augmented grey wolf optimizer, Flood routing, Meta-heuristic algorithms, Non-linear Muskingum model

## Abstract

Flood routing plays a crucial role in prevention of major economic and human losses, which, in this study, has been conducted via both three- and four-constant parameter non-linear Muskingum models for four hydrographs, along with the Grey Wolf Optimizer (GWO) algorithm. Three benchmark examples and a real example (Karun river) were investigated. The routing results of the Karun River revealed that in the estimation of the hydrological parameters using the GWO technique, SSQ became 59294 cms in the three-parameter model, compared to the genetic, artificial bee colony (ABC), simulated annealing (SA) and shuffled frog leaping (SFLA) algorithms, decreasing by 68%, 67%, 56% and 55% in comparison with the best modelings performed. As for the four-parameter model, the amount of reduction was 18% with respect to the particle swarm optimization algorithm.

• The flood routing is carried out by two non-linear Muskingum model.
• The main purpose of this work is to make a comprehensive study between models optimized by AGWO, GWO and other meta-heuristic algorithms.
• In order to compare the results of the GWO algorithm to those of more recent algorithms, the flood routing was performed by using the Augmented Grey Wolf Optimizer algorithm as well.

## Highlights

• The flood routing is carried out by two non-linear Muskingum model.
• The main purpose of this work is to make a comprehensive study between models optimized by AGWO, GWO and other meta-heuristic algorithms.
• In order to compare the results of the GWO algorithm to those of more recent algorithms, the flood routing was performed by using the Augmented Grey Wolf Optimizer algorithm as well.
• The GWO method generated good results on application to four case studies.

## Graphical abstract

**Figure:**
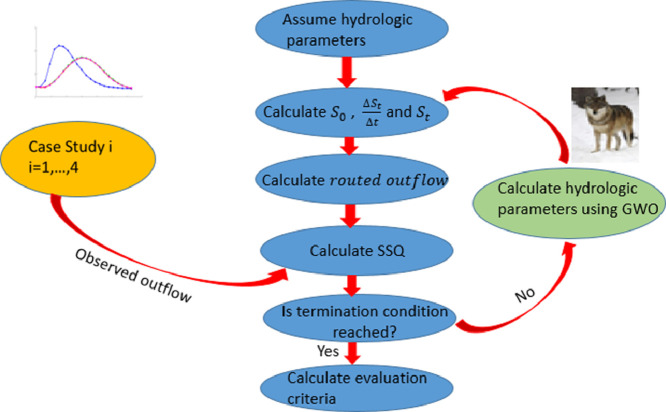

**Specifications table**

**Table:** 

**Subject Area**	Engineering
**More specific subject area**	Meta-heuristic Algorithm, Optimization, Flood Routing
**Method name**	Grey Wolf Optimizer
**Name and reference of original method**	Mirjalili S, Mirjalili SM, Lewis A [22] Grey wolf optimizer. Advances in engineering software, 69, pp.46-61. [32] Augmented grey wolf optimizer for grid-connected PMSG-based wind energy conversion systems. Applied Soft Computing, 69, 504-515.
**Resource availability**	Computer (Intel(R)CoreTMi5 CPU 2.67-GHz) MATLAB software

## Background

Flood routing possesses a number of applications, such as the evaluation of peak flood discharge, the design of hydraulic structures, flood control and management, the drop in property damage as well as in both mental and physical injuries, and the design of flood warning systems. The flood routing methods are divided into two major classes of hydraulic and hydrological methods; within the latter category, the Muskingum method is the most common approach used for flood routing in both linear and non-linear forms. The number of hydrological parameters depends on the type of the Muskingum model employed. Niazkar and Afzali [27] and Kang et al. [16] have proposed different types of the Muskingum model. In addition, various researchers have optimized the hydrological parameters of the Muskingum model through several independent techniques, while the efforts to find more precise methods are ongoing. In the meantime, the meta-heuristic algorithms have been extensively used in the optimal determination of the Muskingum model parameters to date, by means of which it has always been attempted to carry out the flood routing more accurately.

### Literature review

In recent decades, the artificial intelligence-based optimization methods have been widely utilized in the parameter estimation of the Muskingum model, whose outputs have been more accurate than the results of the Lagrange multiplier (LMM), segmented least square (S-LSM) methods. Mohan [24] and Kim et al. [18] implemented the genetic algorithm (GA) and the harmony search (HS) algorithm, respectively; in the former, a new vector is originated from merely two vectors, whereas every single vector in the latter forms a new one. Using the particle swarm optimization (PSO) algorithm, Chu and Chang [6] showed that the PSO outputs are more suitable than those of the GA approach, while the HS algorithm offers more precise results, in comparison with the PSO algorithm. The immune clonal selection algorithm (ICSA) features a higher convergence speed than traditional algorithms, which partly compensates for the defects associated with the GA. In another study, Luo and Xie [19] compared the performance of this algorithm to that of the HS and GA algorithms, and found that the ICSA has a decent speed. Geem [11] and Karahan et al. [17] utilized the parameter setting free-harmony search (PSF-HS) and harmony search-Broyden-Fletcher-Goldfarb-Shanno (HS-BFGS) algorithms, which, owing to their high convergence speed, are used in complex systems. In order to estimate the parameters of their Muskingum model, Niazkar and Afzali [26] adopted the honey-bee mating optimization (HBMO) algorithm, and compared the outputs to the results of 17 other algorithms. Noteworthy in this algorithm is the fast convergence to the optimum value within a broad range of values. This algorithm was subsequently employed by Niazkar and Afzali [27] in conjunction with the generalized reduced gradient (GRG) method for a six-parameter Muskingum model. Hamedi [14] investigated the invasive weed optimization (IWO) algorithm. Moghaddam et al. [23] estimated the Muskingum model parameters by using the PSO algorithm. In order to determine the four hydrological parameters of their Muskingum model, Farahani et al. [9] used the Shark algorithm. Norouzi and Bazargan [28, 29] employed the PSO algorithm to estimate the parameters of Muskingum model.

The GWO algorithm is straightforward programming-wise, which also has a proper accuracy in engineering problems. Investigating the current literature disclosed that the GWO algorithm has not yet been utilized to estimate the parameters of nonlinear Muskingum models. In various publications, this algorithm has either been adopted as single or hybrid with other metaheuristic algorithms so as to improve the precision of the metaheuristic algorithms. Benefiting from the GWO algorithm, Mustafa et al. [25] optimized the parameters of the least-squares support-vector machine (LSSVM), and estimated the water level via the GWO-LSSVM algorithm. In another research work, Marroufpoor et al. [21] used the ANFIS-GWO algorithm for the soil moisture prediction, which was then compared to the artificial neural network (ANN), support vector regression (SVR) and adaptive neuro-fuzzy inference system (ANFIS) algorithms. Hamour et al. [15] adopted the Augmented Grey Wolf Optimizer algorithm (AGWO) to minimize total power losses, and compared their results to those of SPSO (Selective Particle Swarm Optimization) and FEB (Fuzzy Adaptation of Evolutionary Programming). They found that AGWO is an effective and competent method for radial distribution system reconfiguration. Yue et al. [39] developed the GWO algorithm in combination with the FWA (Fireworks Algorithm), named FWGWO, utilized for 16 benchmark functions, whose results were then compared to those of nine other algorithms. Tikhamarine [33] used the GWO algorithm, along with an artificial neural network, for the monthly prediction of the stream flow.

### Innovation and objectives

As mentioned in the previous section, various methods have been proposed over the recent decade to improve the Muskingum model's accuracy. For instance, the optimization algorithm can be employed to estimate the Muskingum model parameters. The use of various algorithms directly affects the objective function.The purpose of this paper is to utilize the AGWO and GWO algorithm in order to calculate the optimal hydrological parameters of two three- and four-parameter Muskingum models, along with comparing the results obtained to those of other algorithms applied in flood routing. The aforementioned algorithms have thus been employed in four case studies, in each of which the outflow hydrograph was developed after having determined the Muskingum model hydrological parameters through the AGWO and GWO algorithms, whose outputs were then compared to those of other algorithms. It has been indicated that this two algorithms determines the hydrological parameters of the three- and four-parameter Muskingum models more precisely, in comparison with all the other algorithms used. It should be noted that although it has outperformed the GWO algorithm in other publications, the results of the AGWO algorithm in all the four case studies are similar to those of the GWO algorithm, with no superiority over each other in terms of the accuracy of the results obtained. This is despite the fact that the GWO algorithm has reached its convergence point in less iteration number and population size, on account of which its outputs were employed in this paper.

## Non-linear Muskingum model

The Muskingum model was originally proposed by the United States Army Corps of Engineers, in 1938, for the flood control in the area along the Muskingum river [1]. Equations (1) and (2) are the nonlinear Muskingum models respectively with three and four parameters (named NL3 and NL4, respectively):

(1) $$S_{t}=K{\left(xI_{t}+\left(1-x\right)O_{t}\right)}^{m}\text{NL}3$$

(2) $$S_{t}=K{\left(xI_{t}^{a}+\left(1-x\right)O_{t}^{a}\right)}^{m}\text{NL}4$$

where *S_t_* = channel storage at time *t* (L^3^); *I_t_* = inflow at time *t* (L^3^/T); *O_t_* = outflow at time *t* (L^3^/T); *k* = time-storage constant for river basin; *x* = a weighting parameter usually in the range of 0 to 0.3; while a and *m* = exponent parameters to consider the nonlinearity effect.

Equation (1) can be obtained in case *a=*1 in Eq. (2). Assuming *a=*1, once again, leads to the outflow magnitude in Eq. (1). Using Eq. (2):

(3) $$O_{t}={\left[\left(\frac{1}{1-x}\right){\left(\frac{S}{k}\right)}^{\frac{1}{m}}-\left(\frac{x}{1-x}\right)I_{t}^{a}\right]}^{\frac{1}{a}}t=2,3,\ldots ,T$$

The continuity equation is as follows:

(4) $$\frac{dS_{t}}{dt}\approx \frac{\Delta S_{t}}{\Delta t}=I_{t}-O_{t}$$

Substituting Eq. (3) into Eq. (4) results in:

(5) $$\frac{\Delta S_{t}}{\Delta t}=I_{t}-{\left[\left(\frac{1}{1-x}\right){\left(\frac{S_{t-1}}{k}\right)}^{\frac{1}{m}}-\left(\frac{x}{1-x}\right)I_{t}^{a}\right]}^{\frac{1}{a}}t=2,3,\ldots ,T$$

The objective function, the minimizer of the sum of squared errors among the routed and observed ouflows (*SSQ*), can be computed as follows:

(6) $$Minimize\;SSQ=\sum_{t}{\left[O_{t}-{\hat{O}}_{t}\left(k,\;x,m,a\right)\right]}^{2}$$

where $O_{t}$: observed outflow and ${\hat{O}}_{t}$: routed outflow.

The flood routing steps are recommended in the following order:

1) A value is assigned to *k, x, m* and *a*;
2) The initial value of *S_t_* is calculated from Eq. (1);
3) Changes in the storage over time (time derivative) is evaluated by using Eq. (5);
4) The storage capacity can then result from the following equation:
  (7) $$S_{t}=S_{t-1}+\frac{dS_{t}}{dt}\Delta t\;t=2,3,\ldots .,T$$
5) The outflow is then computed from Eq. (3) – in many research works, the terms $\left(\frac{I_{t}+I_{t-1}}{2}\right)$ or $I_{t-1}$ has been used instead of *I_t_*;
6) The *SSQ* value is calculated;
7) The magnitudes of *x, k, m* and *a* are updated based on the optimization algorithm;
8) Stages 2-7 should be reiterated until reaching the termination condition [3, 11]

The evaluation indices of *SAD, EQ_p_, ET_p_, MARE* and *VarexQ* have been used as per the following equations in order to assess the performance of the algorithm in this study, as well as to compare it to the previously mentioned methods.

(8) $$SAD=\sum_{t=1}^{T}\left|O_{t}-{\hat{O}}_{t}\right|$$

(9) $$EQ_{P}=\frac{\left|O_{P}-{\hat{O}}_{P}\right|}{O_{P}}$$

(10) $$ET_{P}=\left|T_{P}-{\hat{T}}_{P}\right|$$

(11) $$MARE=\frac{1}{N}\sum_{t=1}^{T}\frac{\left|O_{t}-{\hat{O}}_{t}\right|}{O_{t}}$$

(12) $$VarexQ=\left[1-\frac{\sum_{t=1}^{T}{\left(O_{t}-{\hat{O}}_{t}\right)}^{2}}{\sum_{t=1}^{T}{\left(O_{t}-{\bar{O}}_{t}\right)}^{2}}\right]\times \;100$$

In these equations, $O_{P}$ and ${\hat{O}}_{P}$ represent the peak of observed and routed outflows; $T_{P}$ and ${\hat{T}}_{P}$ denote the peak time at observed and calculated peak outflows, respectively; and ${\bar{O}}_{t}$ is mean of observed outflows. The less the *EQ_p_* is, the more precise will be the model results, while the lower *ET_p_* lead to a more accurate estimation of the peak discharge occurred. Equation (22) can be applied to measure the shape proximity and size of hydrographs [2, 23].

## Grey wolf optimization (GWO) algorithm

The grey wolf algorithm, originally developed by Mirjalili et al. [22], is a subset of swarm intelligence algorithms, which has a hierarchical structure. There are four ranks in each grey wolf pack, the first of which belongs to the alpha male wolves that assert dominance on other groups; the second category comprises the beta male wolves, which enhance the decisions of the first group through giving feedbacks to the alpha male wolves; the next group includes the omega wolves. If a wolf is not an alpha, beta or omega, it is called delta. Delta wolves are supposed to submit to alphas and betas, yet they dominate the omega. The grey wolf algorithm has been established on the basis of pack hunting, which is of interest to grey wolves. The hunting process encompasses three main phases: a) tracking, chasing, and approaching the prey; b) pursuing, surrounding, and exhausting the prey until it stops moving; and c) attacking the prey.

Initially, the population decision variables are randomly developed from the search agents, which are basically the grey wolves, by considering the upper and lower bounds, following which the population of the wolves are sorted from lower to higher fitness based on the value of the fitness function (*SSQ*). The first member in the sorted population is called the alpha wolf, the second one is the beta wolf, and the third one is the delta wolf. The distance of the alpha, beta and delta wolves from the prey is calculated via Eqs. (13-16).

(13) $${\vec{D}}_{a}=\left|{\vec{C}}_{1}.{\vec{X}}_{a}\left(t\right)-\vec{X}\left(t\right)\right|$$

(14) $${\vec{D}}_{\beta }=\left|{\vec{C}}_{2}.{\vec{X}}_{\beta }\left(t\right)-\vec{X}\left(t\right)\right|$$

(15) $${\vec{D}}_{\delta }=\left|{\vec{C}}_{3}.{\vec{X}}_{\delta }\left(t\right)-\vec{X}\left(t\right)\right|$$

(16) $$\vec{C_{i}}=2.{\vec{r}}_{1i}i=1,2,3$$

${\vec{D}}_{\delta }$, ${\vec{D}}_{\beta }$ and ${\vec{D}}_{a}$ are the distances of the alpha, beta and delta wolves from the prey; *t* is the number of iterations; *C* and *X* symbolize the vectors of coefficients and spatial location, respectively; and *r_1i_* are random vectors within the range of [0,1]. The spatial location of the alpha, beta and delta wolves can be updated based on Eqs (17-20).

(17) $${\vec{X}}_{1}\left(t\right)={\vec{X}}_{a}\left(t\right)-{\vec{A}}_{1}.\left({\vec{D}}_{a}\right)$$

(18) $${\vec{X}}_{2}\left(t\right)={\vec{X}}_{\beta }\left(t\right)-{\vec{A}}_{2}.\left({\vec{D}}_{\beta }\right)$$

(19) $${\vec{X}}_{3}\left(t\right)={\vec{X}}_{\delta }\left(t\right)-{\vec{A}}_{3}.\left({\vec{D}}_{\delta }\right)$$

(20) $$\vec{A_{i}}=2.\vec{a}.{\vec{r}}_{2i}-\vec{a}i=1,2,3$$

Where *A* is the vector of coefficients, and *r_2i_* represents the random vectors within the range of [0,1]. The components of the vector *a* decrease linearly from two to zero.

Different spatial locations close to the best option could be achieved by adjusting the vectors *A* and *C*. In order to simulate the hunting process mathematically, it is initially assumed that the alpha, beta and delta wolves have a better knowledge during the hunt. Therefore, the first three optimal solutions are saved, in accordance with which the other search agent wolves (the omegas) are obliged to update their spatial location. The new population members can be evaluated by averaging the new spatial locations as per the following equation:

(21) $${\vec{X}}_{\left(t+1\right)}=\frac{{\vec{X}}_{1}\left(t\right)+{\vec{X}}_{2}\left(t\right)+{\vec{X}}_{3}\left(t\right)}{3}$$

The best member of the new population is introduced as the solution. The process of developing a new population, along with choosing its best member as the solution, continues until meeting the termination condition [22]

The augmented grey wolf algorithm, developed by Qais et al. [32], has augmented the exploration and exploitation process of grey wolf optimizer algorithm. In this algorithm, only alpha and beta wolves are used for hunting and the components of the vector *a* decrease nonlinearly from two to one according to Eq. (22).

(22) $$a=2-\cos\left(rand\right)*\frac{t}{max\_iter}$$

## Results and discussions

**Fig. 1:**
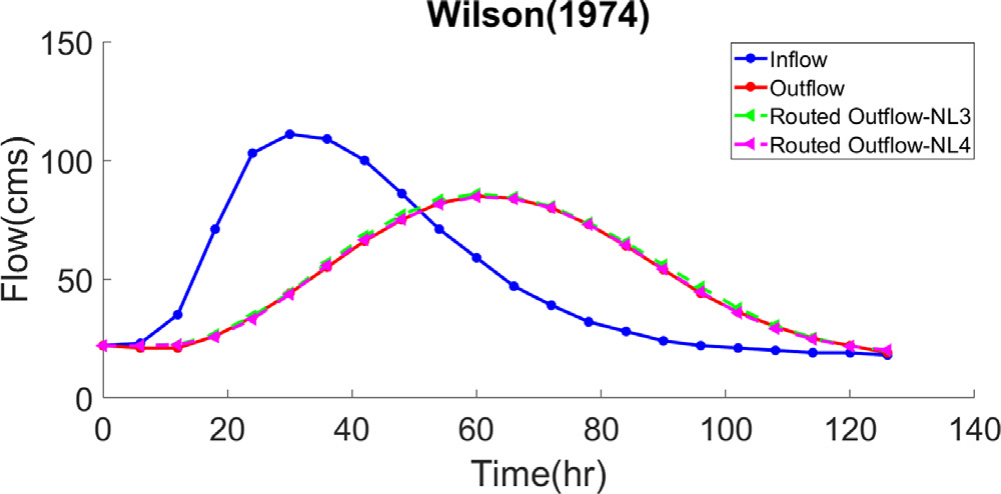
Inflow, observed and routed hydrograph for application of case study 1.

In this research, there are four case studies on which the aforementioned method was tested: a) Wilson River; b) River Wye; c) Viessman and Lewis [36]; and d) Karun River. The flood routing results obtained from the NL3 and NL4 models, as well as those optimized by the GWO algorithm, are as follows:

***Case study 1*** – The Wilson data has been selected as the first example to compare the performance of the nonlinear Muskingum model in this research to those adopted in various studies [37]. With the time step of six hours, the hydrograph considered here has a single peak with an initial inflow rate of 22 cms (Cubic meter per second), which reaches the maximum value of 111 cms in a period of 30 hours, and then diminishes to 18 in 96 hours. Alternatively, the outflows were calculated after having estimated the hydrological parameters in the NL3 and NL4 models via the GWO algorithm. In Table 1, the results of the NL3 and NL4 models are compared to those of twenty NL3 models and five NL4 models. In the former category, the optimal values of *k, x* and *m* as well as the objective function value (*SSQ*) have been calculated as 0.51745, 0.28689, 1.8681 and 36.7679, in that order. In the latter category, the optimal values of *k, x, m* and *a* are 0.1391, 0.2956, 4.0830 and 0.4326, respectively; and the *SSQ* value is equal to 7.6674. It should be noted that the time step of the NL3 and NL4 models was considered to be 6 hours and one hour, respectively. As evidenced in Table 1, the *SSQ* value in the NL3 model optimized by the GWO algorithm has improved compared to the S-LSM, LMM, GA, HJ+CG (Hook-Jeeves Pattern Search (HJ) method in combination with Conjugate Gradient), HJ+DFP (Hook-Jeeves Pattern Search (HJ) method in combination with Davidon-Fletcher-Powell), PSO, ICSA SA, HS, GRG Solver, Evolutionary Solver, DE (Differential Evolution), SFLA, NMS (Nelder-Mead simplex), IICSA (Improved Immune Clonal Selection Algorithm), COBSA (Backtracking Search Algorithm Based Chaotic Orthogonal Design), HS-BFGS, BFGS, AGWO and PSF-HS methods, which is also the case for the *SSQ* value of the NL4 model optimized via the GWO algorithm, in comparison with the PSO, GA-GRG, SFLA-NMS, AGWO and LINGO approaches. Table 2 presents the values of the routed outflows for both models, through which the inflow and outflow hydrographs, together with the routed outflow hydrograph, have been plotted in Fig. 1, in which the close resemblance between the hydrograph routed by means of the GWO algorithm and the observed outflow hydrograph is evident. Using the GWO algorithm, the values of *MARE, VarexQ, EQ_T_, EQ_p_* were optimized for the NL3 and NL4 models, Table 10. Among the various methods applied in the optimal estimation of the hydrological parameters of the NL3 and NL4 models, the GWO algorithm has well reduced the value of *MARE* and *ET_p_*; and the *VarexQ* value is at its maximum, which indicates the good fitness of the proposed model to the observed outflows. Considering the *EQ_P_* value, it can also be declared that the GWO algorithm, similar to some other ones, is well capable of estimating the peak discharge.

**Table 1.** Comparison of best solution with various models for application of case study 1.

Model Type	k	x	m	A	SSQ	Solution Algorithm
NL3						
Gill [12]	0.01	0.25	2.347	-	145.6945	S-LSM
Das [7]	0.0753	0.2769	2.2932	-	130.4872	LMM
Tung [34]	0.0669	0.2685	1.9291	-	49.64	HJ+CG
Tung [34]	0.0764	0.2677	1.8978	-	45.54	HJ+DFP
Mohan [24]	0.1033	0.2813	1.8282	-	38.2363	GA
Chu and Chang [6]	0.1824	0.3330	2.1458	-	36.89	PSO
Luo and Xie [19]	0.0884	0.2862	1.8624	-	36.8026	ICSA
Orouji et al. (2012)	0.08944	0.28735	1.86004	-	36.80	SA
Kim et al. [18]	0.0883	0.2873	1.8630	-	36.7829	HS
Barati [4]	0.0862	0.2869	1.8681		36.77	GRG Solver
Barati [4]	0.0862	0.2869	1.8683	-	36.77	Evolutionary Solver
Xu et al. (2012)	0.5175	0.2869	1.8680	-	36.77	DE
Orouji et al. (2012)	0.08625	0.28692	1.86809	-	36.77	SFLA
Lou et al. [20]	0.0865	0.2870	1.8675	-	36.77	IICSA
Barati [3]	0.0862	0.2869	1.8681	-	36.76	NMS
Yuan et al. [38]	0.0864	0.2869	1.8678	-	36.76	COBSA
Karahan et al. [17]	0.0862	0.278	1.868	-	36.768	HS-BFGS
Present Study	0.5172	0.2868	1.8682	-	36.7680	AGWO
Geem [10]	0.0863	0.2869	1.8679	-	36.7679	BFGS
Geem [11]	0.0863	0.2869	1.8679	-	36.7679	PSF-HS
Present Study	0.5174	0.2869	1.8681	-	36.7679	GWO
NL4						
Moghaddam et al. [23]	0.1659	0.2981	3.6830	0.4689	8.820	PSO
Easa [8]	0.8340	0.2690	4.0790	0.4330	7.670	GA-GRG
Bozorg Haddad et al. [5]	0.834	0.296	4.079	0.433	7.67	SFLA-NMS
Pazoki [31]	0.834	0.296	0.433	4.079	7.67	LINGO
Present Study	0.1399	0.2956	4.0952	0.4310	7.6682	AGWO
Present Study	0.1391	0.2956	4.0830	0.4326	7.6674	GWO

**Table 2.** Comparison of routed outflows obtained from GWO for application of case study 1.

		Observed data (cms)		Computed outflows (cms)	Computed outflows (cms)
j	Time (hr)	I_j_	O_j_	NL3 (Present Study)	NL4 (Present Study)
1	0	22	22	22	22
2	6	23	21	22	22
3	12	35	21	22.4	22.3
4	18	71	26	26.6	25.7
5	24	103	34	34.5	33.1
6	30	111	44	44.2	43.6
7	36	109	55	56.9	55.8
8	42	100	66	68.1	66.5
9	48	86	75	77.1	75.2
10	54	71	82	83.3	81.6
11	60	59	85	85.9	84.7
12	66	47	84	84.5	83.8
13	72	39	80	80.6	80.1
14	78	32	73	73.7	73.0
15	84	28	64	65.4	64.3
16	90	24	54	56.0	54.2
17	96	22	44	46.7	44.6
18	102	21	36	37.8	35.8
19	108	20	30	30.5	29.2
20	114	19	25	25.2	24.7
21	120	19	22	21.7	21.7
22	126	18	19	20.0	20.1

**Fig. 2:**
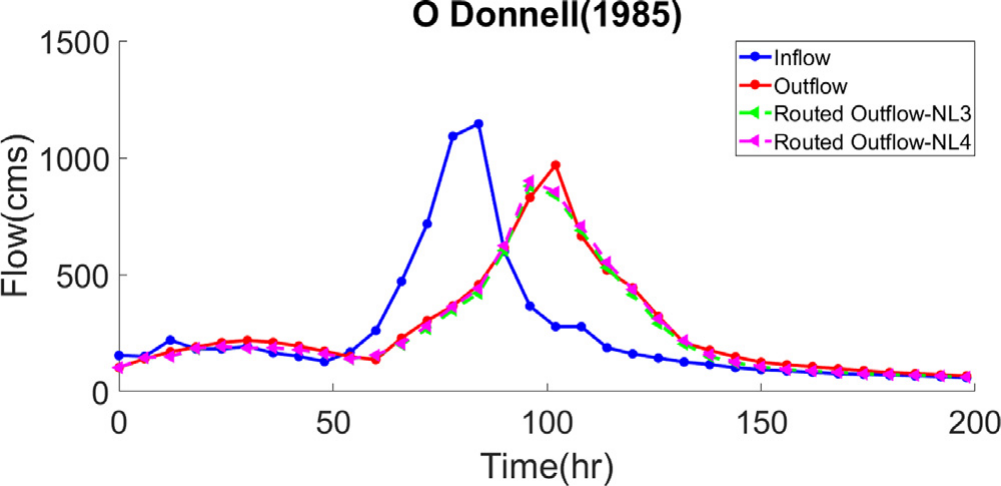
Inflow, observed and routed hydrograph for application of case study 2.

***Case study 2*** – This is a non-smooth flood hydrograph belonging to the River Wye in the United Kingdom, which has been studied in many research works. Table 3 presents the optimal values of the hydrological parameters calculated by the GWO, AGWO, IICSA, HS-BFGS, COBSA, GRG, GA-GRG, SFLA-NMS, PSO and LINGO algorithms. The optimized values are *k=*0.4394, *x=*0.4148 and *m=*1.5915 for the NL3 model as well as *k=*0.1471, *x=0.*4138, *m=*1.3648 and *a=*1.1661 for the NL4 model. In the NL3 model, the GWO-based *SSQ* value decreased by 66%, 16%, 9% and 8% than the IICSA, HS-BFGS, COBSA and GRG algorithms, as well as by 12%, 9%, 9% and 8% for the NL4 model, compared to the GA-GRG, SFLA-NMS, LINGO and PSO algorithms, respectively. Increasing the number of hydrological parameters from three to four has reduced the SSQ value by 11%. For the NL3 and NL4 models, the values of the routed outflows and their corresponding hydrograph can be found in Table 4 and Fig. 2, respectively. Moreover, the performance evaluation indices of the algorithms are shown in Table 10, according to which the *SAD, MARE* and *EQ_p_* parameters have less values than those in other algorithms, whereas the *VarexQ* value has increased. The EQ_p_ was obtained 0.07, being the lowest value among other methods and indicating that the peak discharge was estimated with lower error compared to other methods.

**Table 3.** Comparison of best solution with various models for application of case study 2.

Model Type	k	x	m	a	SSQ	Solution Algorithm
NL3						
Luo et al. [20]	0.001	0.3213	2.2372	-	93735	IICSA
Karahan et al. [17]	0.0792	0.4093	1.5815	-	37944.14	HS-BFGS
Yuan et al. [38]	0.0768	0.4089	1.5861	-	35194.62	COBSA
Hamedi et al. [13]	0.076	0.415	1.59	-	34789.4	GRG
Present Study	0.442	0.414	1.590	-	32018.4	AGWO
Present Study	0.4394	0.4148	1.5915	-	32018.08	GWO
NL4						
Easa [8]	0.437	0.404	1.332	1.197	32299.2	GA-GRG
Bozorg Haddad et al. [5]	0.450	0.414	1.363	1.166	31333.9	SFLA-NMS
Pazoki [31]	0.450	0.414	1.363	1.166	31333.9	LINGO
Moghaddam et al. [23]	0.612	0.401	1.363	1.133	31099.52	PSO
Present Study	0.1494	0.4141	1.3638	1.1653	28558.48	AGWO
Present Study	0.1471	0.4138	1.3648	1.1661	28558.13	GWO

**Table 4.** Comparison of routed outflows obtained from GWO for application of case study 2.

		Observed data (cms)		Computed outflow (cms)	Computed outflow (cms)
j	Time (hr)	I_j_	O_j_	NL3 (Present Study)	NL4 (Present Study)
1	0	154	102	102	102
2	6	150	140	143.8	144.2
3	12	219	169	149.2	149.3
4	18	182	190	182.7	184.3
5	24	182	209	191.4	192.2
6	30	192	218	185.1	185.5
7	36	165	210	187.3	187.4
8	42	150	194	178.4	178.7
9	48	128	172	161.1	161.5
10	54	168	149	139.2	139.9
11	60	260	136	154.6	155.1
12	66	471	228	200.8	204.0
13	72	717	303	267.3	280.7
14	78	1092	366	347.8	362.6
15	84	1145	456	419.1	439.8
16	90	600	615	602.3	623.8
17	96	365	830	879.1	901.0
18	102	277	969	839.0	854.1
19	108	277	665	689.0	707.6
20	114	187	519	530.7	553.2
21	120	161	444	414.3	436.1
22	126	143	321	289.9	310.9
23	132	126	208	202.8	218.0
24	138	115	176	149.8	157.8
25	144	102	148	122.5	125.2
26	150	93	125	104.9	105.8
27	156	88	114	93.5	93.8
28	162	82	106	87.8	87.9
29	168	76	97	81.4	81.5
30	174	73	89	74.9	75.0
31	180	70	81	72.4	72.4
32	186	67	76	69.2	69.2
33	192	63	71	66.1	66.1
34	198	59	66	61.5	61.6

**Fig. 3:**
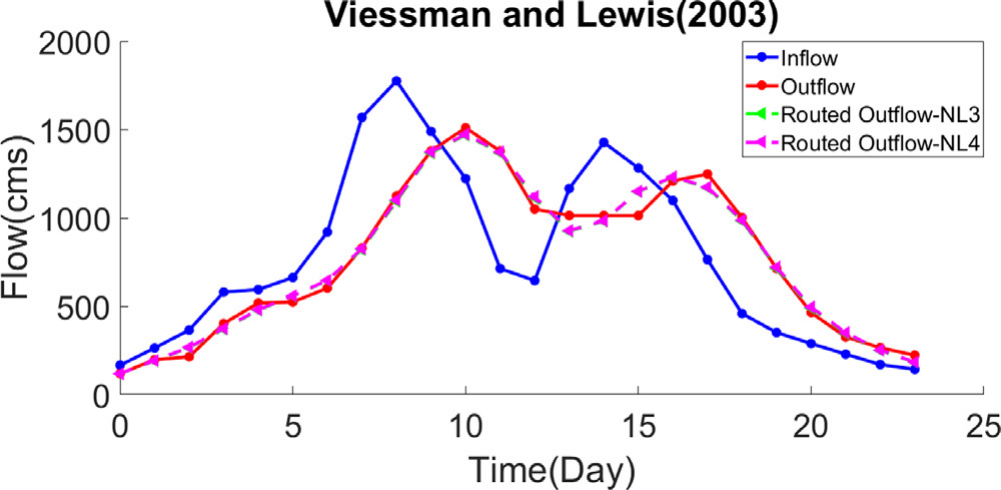
Inflow, observed and routed hydrograph for application of case study 3.

***Case study 3*** – A multi-peak flood hydrograph has been used in this example [36]. Table 5 compares the GWO algorithm to the AGWO, IICSA, GA-GRG, PSO, SFLA-NMS and LINGO algorithms. In the NL3 model, the optimal values of the hydrological parameters estimated by GWO, which reduced the *SSQ* value by 29% than the IICSA algorithm, are *k=*0.4562*, x=*0.4250*, m=*1.2540 and *SSQ=*51742*,* respectively. These were obtained as *k=0.*4535*, x=*0.4266*, m=*1.1987, a=1.0470 and *SSQ=*51527 for the NL4 model. Comparing the current GWO outputs to those of the previous research discloses that this algorithm has diminished the *SSQ* value by 33%, 31%, 30% and 30% with respect to the GA-GRG, PSO, SFLA-NMS and LINGO algorithms. Table 6 presents the routed outflow values and the optimal hydrological parameters for the NL3 and NL4 models. In addition, the hydrographs of the routed and observed outflows and inflows are depicted in Fig. 3. Table 10 shows the evaluation indices calculated via the GWO and five other algorithms. Noting the values of *SAD, EQ_p_, ET_p_, MARE* and *VarexQ* in this example, it can be claimed that GWO estimates the outflows and their peak more efficiently than the other aforementioned algorithms. The *VarexQ* is also higher in this algorithm, which indicates the better agreement between the routed and observed outflows. Thus, the GWO algorithm in this example offers more suitable results in comparison with those in the previous studies.

**Table 5.** Comparison of best solution with various models for application of case study 3.

Model Type	k	x	m	a	SSQ	Solution Algorithm
NL3						
Luo et al. [20]	0.0181	0.2127	1.6287	-	72877	IICSA
Present Study	0.4583	0.4257	1.2535	-	51743	AGWO
Present Study	0.4562	0.4250	1.2540	-	51742	GWO
NL4						
Easa [8]	0.539	0.169	1.648	0.864	76758	GA-GRG
Moghaddam et al. [23]	0.0605	0.1310	4.574	0.3213	74812.30	PSO
Bozorg Haddad et al. [5]	0.077	0.167	1.568	0.921	73379	SFLA-NMS
Pazoki [31]	0.077	0.167	1.568	0.921	73379	LINGO
Present Study	0.4540	0.4264	1.2011	1.0448	51528	AGWO
Present Study	0.4535	0.4266	1.1987	1.0470	51527	GWO

**Table 6.** Comparison of routed outflows obtained from GWO for application of case study 3.

		Observed data (cms)		Computed outflow (cms)	Computed outflow (cms)
j	Time (day)	I_j_	O_j_	NL3 (Present Study)	NL4 (Present Study)
1	0	166.2	118.4	118.4	118.4
2	1	263.6	197.4	191.8	192.9
3	2	365.3	214.1	266.0	267.6
4	3	580.5	402.1	371.5	374.1
5	4	594.7	518.2	478.5	480.5
6	5	662.6	523.9	558.3	559.4
7	6	920.3	603.1	645.5	646.7
8	7	1568.8	829.7	822.1	826.9
9	8	1775.5	1124.2	1095.5	1101.6
10	9	1489.5	1379	1369.4	1374.1
11	10	1223.3	1509.3	1469.7	1473.1
12	11	713.6	1379	1369.5	1373.4
13	12	645.6	1050.6	1115.3	1120.4
14	13	1166.7	1013.7	925.4	928.4
15	14	1427.2	1013.7	982.4	984.4
16	15	1282.8	1013.7	1149.2	1150.8
17	16	1098.7	1209.1	1230.7	1232.1
18	17	764.6	1248.8	1172.8	1174.7
19	18	458.7	1002.4	984.0	987.5
20	19	351.1	713.6	715.8	721.0
21	20	288.8	464.4	490.9	495.6
22	21	228.8	325.6	347.5	350.5
23	22	170.2	265.6	250.5	252.2
24	23	143	222.6	182.8	183.6

**Fig. 4:**
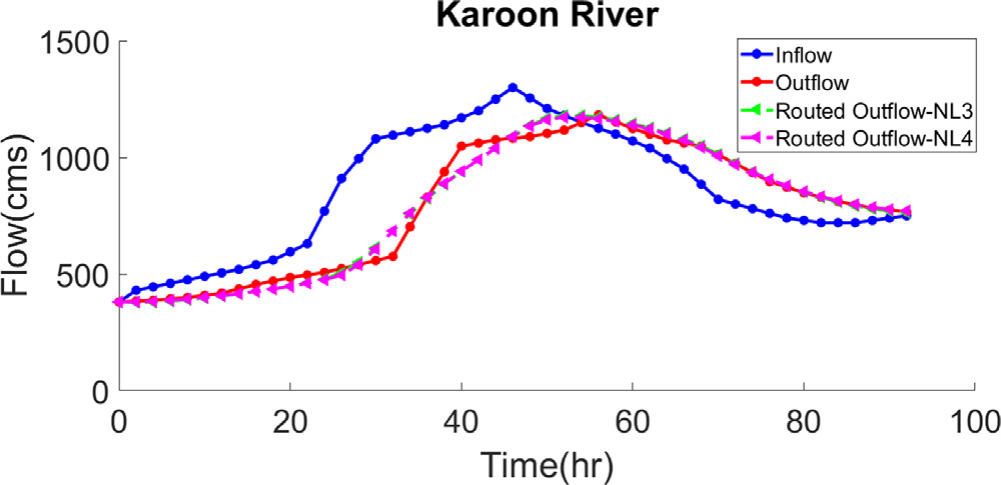
Inflow, observed and routed hydrograph for application of case study 4.

***Case study 4*** – After having been proven in achieving the desired outcomes in the previous examples, the GWO algorithm was adopted for a real river in Iran. Karun is the longest river in the southwest of Iran, to which the Dez river – another important river in the ecosystem – connects as well. The target discharges were recorded by two gauging stations called Godar and Gotvand, with the minimum and maximum inflows being 380 and 1300 cms, respectively. In this single peak hydrograph, the time step is equal to two hours [23], while this was considered to be one hour in the routing process. The parameter estimation results via the GWO, AGWO and GA, ABC, SA, SFLA and PSO algorithms are tabulated in Table 7, in which the optimal value of the hydrological parameters of the NL3 model have been obtained as *k=*14982.7, *x=*0.1457 and *m=*0.1361, with their corresponding *SSQ* as 59294. In this model, the GWO algorithm outperformed the GA, ABC, SA and SFLA algorithms, in terms of substantially reducing the objective function value to the extent that it has decreased by 68%, 67%, 56% and 55%, compared to the mentioned algorithms, respectively. The significant decrease in the objective function is a strong point of the GWO algorithm when compared to a highly capable algorithm such as GA. In the NL4 model, the *SSQ* value of the GWO algorithm declined by 18% than that of the PSO algorithm; and the associated optimal hydrological parameters were evaluated as *k=*41735.3, *x=*0.0762, *m=*0.0210 and *a=*3.393, with the corresponding *SSQ* value of 56698. In the NL3 and NL4 models, in order to assess and further compare the GWO method to other evolutionary algorithms, the performance criteria indices presented in Table 10 were calculated by using the optimal values of the hydrological parameters attained through the GWO algorithm. As evidenced in Table 10, the GWO method has a superior performance; As shown in Table 10, the values of VarexQ and SAD were obtained 98.39 and 1077, respectively. The values of these indexes show that the GWO algorithm had better performance compared to the other methods listed in the table. The inflow and outflow hydrographs of Karun river and the routed outflow hydrograph, as well as the routed outflow values to provide a step-by-step and more accurate comparison, are presented, respectively, in Fig. 4 and Table 8, in which there is an insignificant difference between the routed and observed outflows. It should be added that the model has been fitted well to the observational data.

**Table 7.** Comparison of best solution with various models for application of case study 4.

Model Type	k	x	m	a	SSQ	Solution Algorithm
NL3						
Vafakhah et al. [35]	20.92	0.00001	0.8913	-	182821	GA
Vafakhah et al. [35]	0.7694	0.0001	0.7143	-	177161.4	ABC
Orouji et al. [30]	0.1013	0.2423	1.5500	-	135809.4	SA
Orouji et al. [30]	0.1466	0.2449	1.5000	-	130928.6	SFLA
Present Study	14953	0.1457	0.1362	-	59294	AGWO
Present Study	14982.7	0.1457	0.1361	-	59294	GWO
NL4						
Moghaddam et al. [23]	7740.68	-0.0527	0.9911	0.1786	68790.84	PSO
Present Study	39910	0.0767	0.0227	3.3783	56698	AGWO
Present Study	41735.3	0.0762	0.0210	3.393	56698	GWO

**Table 8.** Comparison of routed outflows obtained from GWO for application of case study 4.

		Observed data (cms)		Computed outflow (cms)	Computed outflow (cms)
j	Time (hr)	I_j_	O_j_	NL3 (Present Study)	NL4 (Present Study)
1	0	380	380	380	380
2	2	430	383.5	378.6	380.1
3	4	445	387	379.4	382.0
4	6	460	393	384.4	386.8
5	8	475	399	390.5	392.5
6	10	490	408.5	397.7	399.3
7	12	505	418	405.8	406.9
8	14	520	436	414.9	415.5
9	16	540	455	424.8	424.9
10	18	560	470	435.8	435.3
11	20	595	485	447.9	446.9
12	22	630	496	462.0	460.5
13	24	770	507	478.1	475.0
14	26	910	523.5	504.3	495.7
15	28	995	540	550.7	540.5
16	30	1080	558	612.8	607.0
17	32	1095	576	684.7	685.0
18	34	1110	702.5	757.8	761.6
19	36	1125	829	824.9	828.7
20	38	1140	938.5	885.5	887.9
21	40	1170	1048	940.1	941.0
22	42	1200	1061.5	990.8	990.5
23	44	1250	1075	1040.0	1039.1
24	46	1300	1082	1090.4	1089.4
25	48	1255	1089	1137.0	1134.3
26	50	1210	1103	1167.2	1161.6
27	52	1180	1117	1179.1	1172.3
28	54	1150	1149.5	1179.2	1172.4
29	56	1125	1182	1172.0	1165.7
30	58	1100	1153	1160.0	1154.6
31	60	1070	1124	1144.6	1139.9
32	62	1040	1099.5	1126.2	1122.0
33	64	995	1075	1104.5	1100.4
34	66	950	1061.5	1078.8	1074.6
35	68	885	1048	1048.4	1043.7
36	70	820	1011	1012.7	1007.2
37	72	800	974	974.1	969.8
38	74	780	935	937.9	936.1
39	76	760	897	906.2	906.0
40	78	740	872.5	877.8	878.6
41	80	730	848	852.1	853.9
42	82	720	829.5	829.4	832.2
43	84	720	811	809.7	813.4
44	86	720	797	793.3	797.9
45	88	730	783	780.3	785.6
46	90	740	774.5	770.8	776.8
47	92	750	766	764.8	771.0

As evidenced in Table 1., Table 2., Table 3., Table 4., Table 5., Table 6., Table 7., Table 8., similar to the GWO algorithm, the AGWO algorithm has estimated the hydrological parameters in all the four case studies with a good accuracy as well, the results of which are also superior to those of the other algorithms presented in Table 1., Table 2., Table 3., Table 4., Table 5., Table 6., Table 7., Table 8..

Table 9 compares these two algorithms in terms of their population size and maximum iteration number. Therefore, the final outputs in this research have been reported based on the GWO algorithm.

**Table 9.** AGWO and GWO parameters.

Model	Case study	Population Size	Algorithm Termination	Solution Algorithm
NL3	Case study 1	300	Maximum Number of Generation(2000)	GWO
	Case study 2	100	Maximum Number of Generation(1000)	
	Case study 3	300	Maximum Number of Generation(300)	
	Case study 4	100	Maximum Number of Generation(500)	
NL4	Case study 1	100	Maximum Number of Generation(1000)	GWO
	Case study 2	100	Maximum Number of Generation(1000)	
	Case study 3	300	Maximum Number of Generation(1000)	
	Case study 4	100	Maximum Number of Generation(1000)	
NL3	Case study 1	1000	Maximum Number of Generation(1000)	AGWO
	Case study 2	1000	Maximum Number of Generation(1000)	
	Case study 3	1000	Maximum Number of Generation(1000)	
	Case study 4	1000	Maximum Number of Generation(1000)	
NL4	Case study 1	1000	Maximum Number of Generation(2000)	AGWO
	Case study 2	1000	Maximum Number of Generation(2000)	
	Case study 3	1000	Maximum Number of Generation(2000)	
	Case study 4	1000	Maximum Number of Generation(2000)

**Table 10.** Comparison of performance criteria indices.

Method	No of Parameters	SAD	EQ_P_	ET_P_	MARE	VarexQ
	Case Study 1					
S-LSM	3	46.40	0.0216	0	0.056	98.83
LMM	3	43.20	0.0000	0	0.055	98.94
HJ+CG	3	25.2	0.0059	0	0.030	99.59
HJ+DFP	3	24.8	0.0035	0	0.030	99.63
GA	3	23.0	0.008	0	0.025	99.70
PSO^a^	3	24.1	0.0007	0	0.030	99.70
ICSA	3	23.40	0.0105	0	0.025	99.69
SA	3	NR	0.0111	0	NR	NR
HS	3	23.40	0.0107	0	0.031	99.63
GRG^b^	3	23.47	0.0106	0	0.025	99.70
Evolutionary Solver	3	23.46	0.0105	0	0.025	99.70
DE	3	23.46	0.0105	0	0.026	99.69
SFLA	3	NR	0.0106	0	NR	NR
IICSA	3	23.45	0.106	0	NR	NR
NMS	3	23.46	0.0106	0	0.025	99.70
COBSA	3	23.47	0.0106	0	0.0253	99.70
HS-BFGS	3	23.40	0.0106	0	0.0251	99.701
BFGS	3	23.46	0.0106	0	0.026	99.69
PSF-HS	3	23.70	0.0105	0	0.026	99.69
AGWO	3	23.46	0.0105	0	0.025	99.70
GWO	3	23.46	0.0106	0	0.025	99.70
PSO^c^	4	10.31	0.0037	0	0.015	99.94
GA-GRG	4	9.77	0.0001	0	0.015	99.93
SFLA-NMS	4	10.31	0.0035	0	0.0151	99.94
LINGO	4	10.31	NR	NR	NR	NR
AGWO	4	10.28	0.0037	0	0.015	99.94
GWO	4	10.31	0.0036	0	0.015	99.94
	Case Study 2					
IICSA	3	1079	0.1702	1	NR	NR
HS-BFGS	3	829	0.1011	6	0.1117	97.7074
COBSA	3	770	0.1014	6	0.0951	97.87
AGWO	3	734.3	0.09	6	0.09	98.06
GWO	3	735.09	0.093	6	0.09	98.06
GA-GRG	4	743.32	0.0784	6	0.1025	98.05
SFLA-NMS	4	732.3	0.0702	6	0.1025	98.1051
LINGO	4	732.3	NR	NR	NR	NR
PSO^c^	4	695.77	0.090	6	0.09	98.12
AGWO	4	673.82	0.070	6	0.086	98.27
GWO	4	673.50	0.070	6	0.09	98.27
	Case Study 3					
IICSA	3	942	0.0549	0	NR	NR
AGWO	3	840.22	0.0260	0	0.059	98.81
GWO	3	840.94	0.0263	0	0.059	98.81
PSO^c^	4	NR	0.0358	0	0.067	98.27
SFLA-NMS	4	1034	NR	NR	NR	NR
LINGO	4	1034	NR	NR	NR	NR
AGWO	4	825.5	0.0241	0	0.058	98.81
GWO	4	824.22	0.0240	0	0.058	98.81
	Case Study 4					
SA	3	1923.9	0.0123	0	0.0543	96.15
SFLA	3	1881.2	0.011	0	0.0527	96.28
AGWO	3	1117.1	0.0023	2	0.03	98.32
GWO	3	1117.1	0.0023	2	0.03	98.32
PSO^c^	4	1067.1	0.0122	2	0.03	98.05
AGWO	4	1077	0.0081	2	0.032	98.39
GWO	4	1077	0.0081	2	0.032	98.39

a Chu and Chang(2009)

b Barati (2012)

c Moghaddam et al. [23] NR means the performance evaluation criteria and the computed outflows were not reported.

## Conclusion

Given the significance of flood forecasting and its role in designing the flood-resistant hydraulic structures, as well as the measures required for flood control and management, the flood routing is of special interest to the researchers in this field, to the extent that it has always been attempted to achieve this imperative quickly and accurately. In this research, the flood routing was conducted by using two nonlinear Muskingum models with three and four constant parameters. In order to estimate the parameters of both models, the grey wolf optimization (GWO) algorithm was utilized in optimization problems because of its efficiency, which has not yet been applied in flood routing processes; and it was thus used for three case studies and a real example in Iran (Karun River), in each of which its performance has been compared to that of other evolutionary algorithms, in terms of the *SSQ, SAD, EQ_p_, ET_p_, MARE* and *VarexQ* values. It was revealed, in all the four case studies, that the GWO algorithm is well capable of optimally estimating the Muskingum model parameters, whose application in flood routing problems is strongly recommended. Eventually, a comparison between the results of the AGWO and GWO algorithms has been conducted in four case studies. The aforesaid algorithms in the four case studies have reduced the *SSQ* value up to 55% than the minimum *SSQ* value, which can be implemented to optimize the hydrological parameters of other forms of the Muskingum equation, whether single or combined with other algorithms such as GA and PSO.

## Declaration of Competing Interest

The authors declare that they have no known competing financial interests or personal relationships that could have appeared to influence the work reported in this paper.
